# New Insights into the Mechanism of Action of Soluble Klotho

**DOI:** 10.3389/fendo.2017.00323

**Published:** 2017-11-17

**Authors:** George D. Dalton, Jian Xie, Sung-Wan An, Chou-Long Huang

**Affiliations:** ^1^Department of Medicine, Division of Gastroenterology, Duke University Medical Center, Durham, NC, United States; ^2^Department of Internal Medicine, Division of Nephrology and Hypertension, University of Iowa Carver College of Medicine, Iowa City, IA, United States

**Keywords:** klotho, FGF23, lipid rafts, aging, TRPC6, sialidase, IGF-1, heart disease

## Abstract

The *klotho* gene encodes a type I single-pass transmembrane protein that contains a large extracellular domain, a membrane spanning segment, and a short intracellular domain. Klotho protein exists in several forms including the full-length membrane form (mKl) and a soluble circulating form [soluble klotho (sKl)]. mKl complexes with fibroblast growth factor receptors to form coreceptors for FGF23, which allows it to participate in FGF23-mediated signal transduction and regulation of phosphate and calcium homeostasis. sKl is present in the blood, urine, and cerebrospinal fluid where it performs a multitude of functions including regulation of ion channels/transporters and growth factor signaling. How sKl exerts these pleiotropic functions is poorly understood. One hurdle in understanding sKl’s mechanism of action as a “hormone” has been the inability to identify a receptor that mediates its effects. In the body, the kidneys are a major source of sKl and sKl levels decline during renal disease. sKl deficiency in chronic kidney disease makes the heart susceptible to stress-induced injury. Here, we summarize the current knowledge of mKl’s mechanism of action, the mechanistic basis of sKl’s protective, FGF23-independent effects on the heart, and provide new insights into the mechanism of action of sKl focusing on recent findings that sKl binds sialogangliosides in membrane lipid rafts to regulate growth factor signaling.

## Discovery of the Aging-Suppressor Gene *klotho*

For decades, scientists have searched for genes that regulate lifespan. In 1997, one such gene was identified in a transgenic mouse strain whose mutation resulted in a syndrome resembling premature aging that included shortened lifespan, growth retardation, vascular calcification, genital atrophy, emphysema, and osteomalacia (1). The gene was named *klotho*, which in Greek mythology is one of the three goddesses of fate who spins the thread of life (1). The aging phenotypes were observed exclusively in mice that were homozygous for *SLC9A1* transgene insertion into the 5′ flanking region of the *klotho* gene, which resulted in a severe hypomorphic *klotho* allele (*kl/kl*). Since the discovery, *klotho* attracted considerable scientific interest due to its role in aging suppression. Abundant evidence has accumulated during the past two decades that supports the association between *klotho* and senescence. For instance, transgenic mice that overexpress *klotho* exhibit an extended lifespan compared with wild-type (WT) mice which has been attributed, at least partly, to *klotho*-induced resistance to insulin signaling and oxidative stress (2, 3). In humans, total Klotho protein levels decline with age in serum, while single nucleotide polymorphisms have been identified in the *klotho* gene that correlates with reduced longevity and the pathophysiology of age-related disorders such as osteoporosis, coronary artery disease, and stroke (4–8). Finally, gene profile analyses have demonstrated that *klotho* expression is decreased in aged brain white matter in rhesus monkeys indicating a role for *klotho* as a lifespan gene in the nervous system (9).

The *klotho* gene encodes a 130 kDa type I single-pass transmembrane glycoprotein called α-Klotho that contains a short intracellular domain composed of 10 amino acids and an extracellular (EC) domain containing two internal repeats (KL1 and KL2) that are both approximately 450 amino acids long with sequence homology to family 1 β-glycosidases (1). α-Klotho differs from family I glycosidases due to the absence of two conserved glutamic acid residues in its KL1 and KL2 regions that are important for the catalytic activity of this enzyme family (1, 10–12). α-Klotho has been reported to exhibit sialidase and β-glucuronidase activities (13–16). Three primary isoforms of the α-Klotho protein have been identified as follows: (1) the full-length transmembrane form (mKl), (2) a shed soluble form [soluble klotho (sKl)], and (3) a secreted truncated form that is produced by alternative splicing of *klotho* mRNA and consists of KL1 only (17, 18). In the EC space, the secreted truncated form is presumably much less abundant relative to the shed form.

mKl associates with fibroblast growth factor receptors (FGFRs) to form coreceptors for the bone-derived phosphaturic hormone FGF23 (19, 20). sKl is produced when the mKl EC domain is shed from the cell surface into the blood, urine and cerebrospinal fluid following proteolytic cleavage of mKl near the juxtamembrane region by the metalloproteinases ADAM10 and ADAM17 (21–25). Following its release from the cell membrane, circulating sKl exerts its biological effects on distant organs or tissues. Gene and protein expression analyses show that α-Klotho is abundantly expressed in rodents and humans in the kidney and the choroid plexus of the brain, and to a lesser extent in areas such as the parathyroid gland, thyroid gland, pancreas, and sex organs (1, 26–28). Finally, the *klotho* gene family includes two additional family members β-Klotho and γ-Klotho (29, 30). Like α-Klotho, β-Klotho and γ-Klotho are type I single-pass transmembrane proteins that share sequence homology to family 1 β-glycosidases but lack dual conserved glutamic acid residues that are essential for enzymatic glycosidase activities (29, 30). β-Klotho is expressed mainly in liver, adipose tissue, and pancreas, whereas γ-Klotho is expressed in the kidney and skin (29, 30). FGF19 and FGF21 require β-Klotho as a coreceptor to bind FGFRs and activate FGF signaling pathways that regulate bile acid synthesis and energy metabolism (31).

## Functions and Mechanism of Action of sKl

Binding of FGF23 to mKl-FGFR coreceptors plays critical roles in vitamin D, calcium, and phosphate metabolism (19, 20, 32). Homozygous hypomorphic *kl/kl* mice have severe hypervitaminosis D, hypercalcemia, hyperphosphatemia, and extensive tissue calcification (32, 33). Dietary vitamin D or phosphate restriction rescues growth retardation and premature death in *klotho^−/−^* mice, validating that function of mKl as a coreceptor for FGF23 is critical for normal vitamin D and mineral metabolism, as well as growth and lifespan (32, 33). By contrast, the function and mechanism of action of sKl are less clear. Several recent studies have provided important information to advancing our understanding of the function and mechanism of action of sKl. In this review, we will summarize the current knowledge of pleiotropic functions of sKl and discuss recent studies that decipher the molecular mechanisms of action of sKl by identifying its receptors. Finally, we will review the cardioprotective function of sKl to illustrate an important function of sKl independently of the FGFR–FGF23 axis.

### sKl Can Function As a Circulating Hormone

α-Klotho is predominantly expressed in the kidney and brain (1). However, *klotho^−/−^* mice exhibit functional defects in cells that do not express α-Klotho suggesting that circulating sKl can function as a hormone to act at a distance. Overexpression of the *klotho* gene extends lifespan in the mouse (2). The antiaging effects of α-Klotho have been attributed to inhibition of insulin-like signaling, which is an evolutionarily conserved mechanism for suppressing aging (34). *In vitro* studies have demonstrated that sKl suppresses autophosphorylation of insulin/IGF-1 receptors and downstream signaling events that include tyrosine phosphorylation of insulin receptor substrates (IRS) and phosphoinositide 3-kinase (PI3K) p85 association with IRS proteins (2). In addition, inhibition of insulin/IGF-1 signaling alleviated aging-like phenotypes in *klotho^−/−^* mice (2). sKl-mediated inhibition of insulin/IGF-1/PI3K signaling may suppress aging by inducing resistance to oxidative stress. The insulin/IGF-1/PI3K pathway is linked to oxidative stress *via* the FoxO forkhead transcription factors (FOXOs) that are downstream targets of insulin-like signaling that regulate aging (34). Inhibition of insulin-like signaling results in FOXO activation and the upregulation of genes that encode antioxidant enzymes, such as mitochondrial manganese superoxide dismutase (MnSOD), that is important for removing reactive oxygen species and reducing oxidative stress (35). Studies have revealed treatment of cultured cells with sKl reduces lipid oxidation and apoptosis induced by the superoxide-generating herbicide paraquat by blocking insulin-mediated inhibition of FOXO which promoted FOXO activation and nuclear translocation (3). Nuclear FOXO was shown to bind to the MnSOD gene promoter and increase MnSOD protein levels (3). Insulin-induced FOXO phosphorylation/inactivation was enhanced in *klotho^−/−^* mice and attenuated in transgenic mice that overexpress α-Klotho (3). Compared with WT mice, α-Klotho-overexpressing transgenic mice exhibited increased MnSOD protein levels in muscles, reduced urinary 8-OHdG levels (*in vivo* marker of oxidative DNA damage), and enhanced survival following a challenge with a lethal dose of paraquat (3).

In addition to the insulin/IGF-1 pathway, sKl has been shown to confer cytoprotective effects through other antioxidative pathways. For instance, vascular calcification is a phenotype observed in mice homozygous for a hypomorphic *klotho* allele (*klotho^−/−^)* (1). Oxidative stress contributes to the progression of vascular calcification by inducing apoptosis and senescence in vascular endothelial cells. sKl is considered to act as a hormone in the vasculature where it is continuously exposed to vascular endothelial cells. Studies have demonstrated that sKl reduces H_2_O_2_-induced apoptosis and senescence in human umbilical vascular endothelial cells (HUVECs) by inhibiting the caspase 3/caspase 9 and p53/p21 pathways (36). The antiapoptotic and anti-senescence effects of sKl in HUVECs may be mediated by the mitogen-activated protein kinase (MAPK)/extracellular signal-regulated kinase (ERK) pathway, while sKl has also been shown to exert antioxidative effects in HUVECs by inducing MnSOD expression *via* activation of the cAMP/protein kinase A (PKA) pathway (37, 38). In addition to endothelial cells, *klotho* gene transfer attenuated angiotensin II-induced superoxide production, oxidative damage, and apoptosis in vascular smooth muscle cells by stimulating cAMP/PKA-mediated suppression of Nox2 NADPH oxidase protein expression (39). *In vitro* and *in vivo* studies have also demonstrated that sKl protects the lung against oxidative damage. In cultured lung epithelial cells, sKl protected the cells from hyperoxic and phosphotoxic injury by increasing cell oxidative capacity *via* induction of nuclear factor erythroid-derived 2-related factors 1 and 2 (Nrf1/2) transcriptional activity (40). In an acute hyperoxic lung injury animal model, injection of sKl-containing medium into rat peritoneum alleviated oxidative damage and interstitial edema and stimulated an increase in total antioxidant capacity (40). Finally, studies indicate α-Klotho acts as an antioxidant effector in liver and brain by modulating the reactive oxygen species-sensitive apoptosis signal-regulating kinase 1/p38 MAPK pathway (41, 42).

Elevated plasma sKl levels are independently associated with a decreased likelihood of cardiovascular disease (CVD) in humans (43). sKl may be a risk factor for CVD based on studies that have demonstrated endothelial dysfunction is inversely correlated with α-Klotho expression (1, 44). Endothelial dysfunction plays a role in the development of atherosclerosis and is characterized by reduced bioavailability of NO, impaired endothelium-dependent vasorelaxation, increased endothelial permeability, increased oxidative stress, and increased expression of adhesion molecules, pro-inflammatory, and pro-thrombotic factors (45, 46). sKl may exert vasoprotective effects on the endothelium and reduces endothelial dysfunction by regulating NO availability. Studies have shown that NO production and vasodilation are impaired in *klotho^+/−^* mice, whereas endothelial function can be restored in *klotho^+/−^* mice by parabiosis with WT mice (44, 47). In Otsuka Long-Evans Tokushima Fatty rats, an experimental animal model of atherosclerosis, adenovirus-mediated *klotho* gene delivery ameliorated vascular endothelial dysfunction, increased NO production, reduced elevated blood pressure, and prevented medial hypertrophy and perivascular fibrosis (48). Mechanistic investigations using HUVECs have demonstrated that sKl upregulates NO production *via* a cAMP-dependent pathway (37). The cAMP–PKA pathway is known to contribute to activation of endothelial NO synthase and increased NO production in coronary arteries (49–51).

Soluble klotho also prevents endothelial dysfunction by maintaining endothelial integrity and protecting against vascular permeability. In endothelial cells, calcium regulates numerous functions including proliferation, migration, and apoptosis (52). Studies report that sKl binds the transient receptor potential canonical 1 (TRPC1) calcium-permeable channel and vascular endothelial growth factor receptor 2 to strengthen their association and cause their cointernalization which regulates the expression level of TRPC1 on the plasma membrane (53). This allows sKl to tightly regulate VEGF-stimulated calcium entry and hyperactivity of calcium-dependent proteases in endothelial cells which maintains endothelial integrity (53). In support of sKl’s role in maintaining endothelial integrity, the vascular endothelium is hyperpermeable in *klotho^−/−^* mice, believed due to increased TRPC1 expression and TRPC1-mediated calcium influx, hyperactivation of calcium-dependent calpain/caspase 3, and increased apoptosis and endothelial damage (53). Finally, a growing body of evidence indicates vascular inflammation plays an important role in endothelial dysfunction. Pro-inflammatory molecules, such as tumor necrosis factor-α (TNF-α), upregulate adhesion molecules on the surface of endothelial cells (54, 55). Moreover, studies have demonstrated that the expression of the adhesion molecules ICAM-1 and VCAM-1 are increased in animals with inflammation and in human atherosclerotic plaques (54). Recombinant sKl inhibited TNF-α-induced expression of ICAM-1 and VCAM-1 on HUVECs (56). In addition, sKl blocked TNF-α-induced NF-κB activation in HUVECs, which is significant because NF-κB is a transcription factor that regulates ICAM-1 and VCAM-1 expression (56). Thus, sKl may maintain endothelial integrity by regulating the expression of endothelial cell inflammatory mediators such as adhesion molecules and NF-κB.

Tumor suppressor genes regulate cell proliferation and inhibit tumor development. *Klotho* may be a tumor suppressor in a wide range of malignancies that include breast cancer, cervical cancer, pancreatic cancer, melanoma, gastric cancer, colorectal cancer, lung cancer, liver cancer, renal cell carcinoma, and ovarian cancer (57–67). In all of these cancers, *klotho* expression was reduced in tumor tissue compared with normal tissue. Epigenetic modifications, such as DNA methylation and histone modifications, often play an important role in regulating the expression of tumor suppressor genes (68). Promoter methylation and histone deacetylation have been found to be epigenetic silencing mechanisms of *klotho* expression in multiple types of cancer (58, 61–63, 65). In addition, microRNAs appear to play a role in cancer progression by targeting *klotho* and regulating its expression (68–70). The reduction of *klotho* expression in malignant tissue suggests that α-Klotho has anticancer effects. Studies by re-expression of *klotho* in cancer cells revealed that sKl acts as a tumor suppressor by inhibiting multiple signaling pathways that include the insulin/IGF-1 pathway, FGF pathway, Wnt signaling pathway, and transforming growth factor-β1 (TGF-β1) pathway.

The insulin/IGF-1 signaling pathway plays an important role in cell proliferation, apoptosis, and cancer (71, 72). Insulin and IGF-1 binding to their receptors activates IRS proteins leading to activation of PI3K/Akt or MAPK/ERK1/2 cell signaling pathways, which play a role in the normal development and maintenance of tissues. Dysregulation of these pathways can lead to tumor development and progression. α-Klotho acts as a tumor suppressor by inhibiting insulin/IGF-1 signaling in breast cancer, lung cancer, pancreatic cancer, gastric cancer, liver cancer, colon cancer, and ovarian cancer (57, 59, 65, 73–76). Overexpression of α-Klotho or treatment with sKl inhibits insulin/IGF-1-mediated downstream effectors IRS-1, Akt1, and ERK1/2 in cancer cells (57, 59, 65, 67, 73–76). The tumor suppressive activity has been attributed to its KL1 domain (59, 76).

Defects in the regulation of the Wnt signaling pathway also cause cancer (77). Wnt signaling is initiated when secreted Wnt ligands activate transmembrane receptors that promote the translocation of β-catenin to the nucleus where it induces the activity of transcription factors such as TCF and LEF (77). The activation of gene transcription by β-catenin leads to the synthesis of genes, such as *c-myc* and *cyclin D1*, that cause cancer cell growth and invasiveness (78). sKl is a Wnt antagonist that binds to multiple Wnt ligands and inhibits their activation of Wnt signaling (79). sKl inhibition of the Wnt signaling pathway has been shown to reduce cancer cell invasiveness, proliferation, and viability, while it increased cancer cell apoptosis (60, 64, 80). Biochemical evidence has shown that sKl reduces Wnt5A and Wnt3A expression and internalization in melanoma and lung cancer cells, which downregulates Wnt–β-catenin signaling and expression of the Wnt target genes *c-myc* and *cyclin D1* (60, 64). In melanoma cells, sKl reduced cell invasiveness by inhibiting Wnt5A stimulation of μ-calpain-mediated cleavage of Filamin A (60). TGF-β1 signaling pathway plays an important role in cancer metastasis (81), and sKl suppressed TGF-β1-induced epithelial-to-mesenchymal transition to inhibit renal fibrosis and cancer metastasis in mice (82). *In vitro* studies revealed sKl binds the type II TGF-β receptor to reduce TGF-β1 binding which inhibited receptor activation, Smad3 phosphorylation, and Smad3 transcriptional activity (82). Finally, α-Klotho acts as a tumor suppressor by modulating the FGF signaling pathway. Basic FGF-mediated ERK1/2 phosphorylation and activation of the FGF pathway inhibit colony formation in breast cancer cells (57). Overexpression of α-Klotho enhanced bFGF-mediated ERK1/2 phosphorylation and FGF pathway activation in these cells (57). In pancreatic cancer cells, overexpression of α-Klotho or the α-Klotho KL1 domain reduced bFGF-mediated phosphorylation of Akt and ERK1/2 and cancer cell growth (59).

### Identification of Membrane Lipid Rafts and Gangliosides As Receptors for sKl

**Figure 1:**
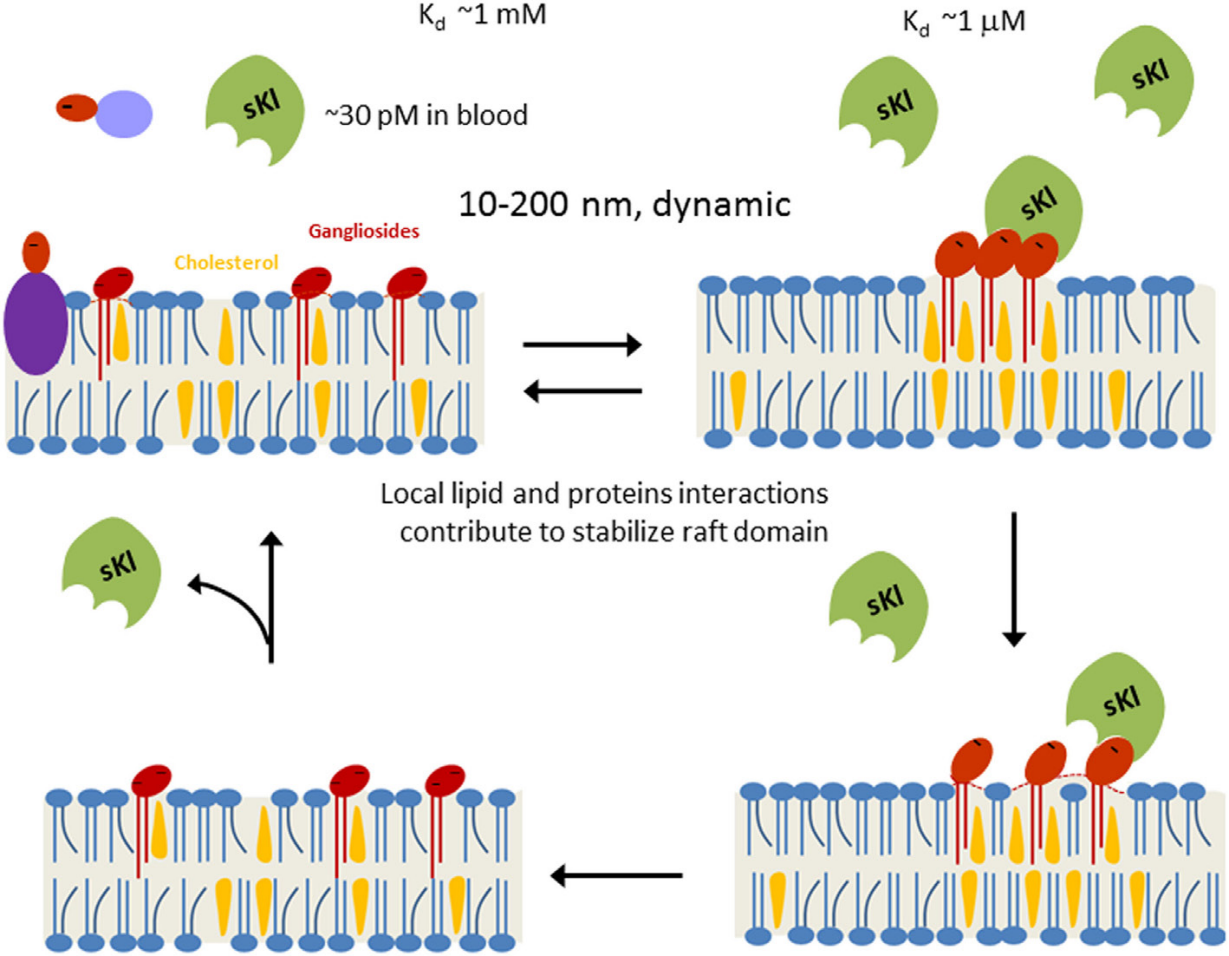
Working model for soluble klotho (sKl) regulation of lipid rafts. Lipid rafts are highly dynamic cholesterol- and sphingolipid-rich membrane microdomains (10–200 nm in size). Formation of lipid rafts is governed by physicochemical properties of lipids and stabilized by local lipid–protein and protein–protein interactions. α2,3-Sialyllactose (dark-red ovale) is a common glycan motif present in many secreted glycoproteins, membrane glycoproteins, and glycolipids such as gangliosides. Due to low circulating concentration (~30 pM) and low binding affinity (*K*_d_ ~1 mM), sKl does not bind to isolated α2,3-sialyllactose significantly. Clustering of α2,3-sialyllactose-containing gangliosides in lipid rafts enhances the “apparent” binding affinity for the likely multivalent sKl. Binding of sKl to gangliosides decreases the formation of rafts. sKl is likely multivalent for binding sialyllactose because each sKl contains homologous KL1 and KL2 domains and it likely exists as dimers (86).

As a “hormone,” sKl regulates multiple signaling pathways to elicit pleiotropic cellular effects. However, the mechanism of action of hormonal sKl remains poorly understood in part because membrane receptors for sKl have not been identified. Recent studies have shed light on this gap in knowledge and identified monosialogangliosides GM1 and GM3 present in lipid rafts as receptors for sKl (83). sKl co-migrated with lipid raft fractions in sucrose gradient ultracentrifugation experiments indicating sKl’s affinity for lipid rafts (83). Förster resonance energy transfer (FRET) and fluorescence lifetime imaging microscopy studies demonstrated sKl alters lipid organization and decreases membrane order within rafts (83). Studies have shown that inhibition of PI3K-dependent TRPC6 function underlies cardioprotection by sKl (84). sKl also selectively downregulated growth factor-driven PI3K/Akt signaling and TRPC6 channel function in lipid rafts, but not in non-lipid raft regions (83). *In vitro* binding assays and competition experiments using TRPC6-based functional assays identified α2,3-sialyllactose in the glycan of GM1 and GM3 gangliosides as the minimal motif required for sKl binding and regulation of TRPC6 in lipid rafts (83). Furthermore, these assays demonstrated that sKl affinity is 300-fold greater for clustered α2,3-sialyllactose compared with free α2,3-sialyllactoses which supports the notion that lipid rafts enriched in α2,3-sialyllactose-containing GM1 and GM3 gangliosides are effective targets for physiologically low circulating concentrations of sKl (~30 pM) (83). Sialylated glycans bind specifically to a number of glycan-binding proteins, but these binding interactions tend to be of low affinity. The formation of glycan clusters is a common mechanism that generates high affinity biologically relevant binding sites for multivalent glycan-binding proteins (85). Moreover, sKl is likely multivalent due to the fact that sKl forms dimers and each unit contains two highly homologous KL1 and KL2 domains with potential glycan-binding activity (86). The multimeric nature of sKl and the clustering of gangliosides likely explain why circulating sKl preferentially targets GM1 and GM3 clustered in lipid rafts rather than un-clustered GM1 and GM3 present in non-raft membranes or isolated α2,3-sialyllactose residues present in glycoproteins (Figure 1). The idea of sKl specifically binding lipid rafts was further supported by FRET experiments in live cells that showed sKl selectively interacts with lipid raft-associated GM1, as well as permeation experiments using hexyltriphenylphosphonium (C6TPP) showing sKl has no effect on disordered membranes (i.e., non-lipid raft membrane regions) (83). The *in vivo* relevance of these findings was confirmed by the discovery that raft-dependent PI3K signaling is upregulated in *klotho^−^*^/^*^−^* mouse hearts compared with WT mouse hearts (83). By contrast, PI3K signaling in non-raft membranes is not different between WT and *klotho^−/−^* mouse hearts (83).

To further support the notion that sKl binds sialogangliosides in lipid rafts to regulate TRPC6 and cardioprotection, the investigators determined a modeled structure of sKl by homology modeling and used docking protocols to examine the potential binding sites in sKl for α2,3-sialyllactose (87). It was shown that Arg^148^, His^246^, and the ^465^EWHR^468^ motif found in the KL1 domain of sKl are important for binding α2,3-sialyllactose (87). Binding experiments using biolayer inferometry showed the KL1 domain alone indeed binds α2,3-sialyllactose with a *K*_d_ value that is similar to that reported for the entire ectodomain of sKl (83, 87). Finally, purified recombinant KL1 domain inhibits TRPC6 in cultured cells and protects against stress-induced cardiac hypertrophy in mice (87). Overall, these studies provide compelling evidence supporting that sialogangliosides GM1 and GM3 and lipid rafts can serve as membrane receptors for sKl.

### sKl Functions As an Enzyme to Regulate Ion Channels/Transporters

Binding of FGF23 to FGFRs and the coreceptor mKl inhibits the synthesis of 1,25(OH)_2_–vitamin D (32). Elevated 1,25(OH)_2_–vitamin D causes hypercalcemia in *klotho^−^*^/^*^−^* mice (88). In addition, sKl plays an important role in calcium homeostasis by regulating the transient receptor potential vanilloid type 5 (TRPV5) calcium channel located at the apical surface of the distal convoluted and connecting tubules that is responsible for calcium reabsorption in the distal nephron (89–91). sKl directly increases renal calcium reabsorption by enhancing cell-surface abundance of TRPV5. An early study demonstrated sKl increases TRPV5 cell-surface abundance by modifying *N*-glycan chains of TRPV5 (14). Subsequent investigations sought to identify the specific TRPV5 sugar residues that were modified by sKl and how *N*-glycan modification led to TRPV5 accumulation in the plasma membrane. Structurally, the *N*-glycan chains of TRPV5 can consist of as many as four branches (92, 93). Individual *N*-glycan branches are initiated by *N*-acetylglucosamine addition to mannose residues followed by galactose addition to form *N*-acetyllactosamine (LacNAc) (93). Galactoses can be capped with sialic acids in a reaction catalyzed by α2,3- and α2,6-sialylytransferases (94–96). sKl increases cell-surface abundance of TRPV5 by acting as a sialidase and specifically removing terminal α2,6-linked sialic acids from TRPV5 *N*-glycan chains (15). Galectins are a family of galactose-binding lectins present extracellularly on the cell surface as well as inside the cell (97, 98). Galectin-1 binds LacNAc, but not α2,6-sialylated LacNAc (99). sKl removal of terminal α2,6-sialic acids from TRPV5 *N*-glycan chains exposes LacNAc residues which bind EC galectin-1 present on the cell surface (15). The binding of galectin-1 to TRPV5 prevents endocytosis and leads to channel accumulation on the cell membrane (15). In general, the affinity for binding galectin-1 is enhanced by the polymeric structure of LacNAc in the *N*-glycan chains. Functional TRPV5 channels have a tetrameric stoichiometry which increases *N*-glycan number, polymeric LacNAc, and the affinity of TRPV5 for galectin-1 (100, 101).

In addition to TRPV5, sKl regulates other ion channels and transporters in the kidney by modifying their *N*-glycan chains. sKl increases the cell-membrane abundance of renal outer medullary potassium channel 1 (ROMK1) by removing terminal α2,6-sialic acids from *N*-glycans of the channel (16). Like TRPV5, removal of α2,6-sialic acids exposes underlying LacNAc which binds galectin-1 to prevent ROMK1 endocytosis leading to accumulation of functional channel on the plasma membrane (16). Together with the finding that sKl regulates membrane lipid rafts by binding sialogangliosides, targeting sialic acids may be a general mechanism for pleiotropic actions of sKl. How sKl appears in the urinary lumen remains unclear. Possibilities include shedding of mKl present in the apical membrane of tubular epithelial cells (if present) or *via* transcytosis from the systemic circulation across the proximal and distal renal tubules (102). Finally, it should be noted that apically localized mKL could conceivably act on TRPV5 or ROMK1 *in situ*.

## FGF23-Independent Cardioprotection by sKl

Cardiac hypertrophy is highly prevalent in patients with chronic kidney disease (CKD) and associated with increased mortality risk (103–106). Conventional risk factors, such as hypertension and volume overload, play important roles in the development of cardiac hypertrophy in CKD (104, 106–108). In addition, multiple CKD-specific risk factors increase the likelihood of cardiac hypertrophy including elevated circulating FGF23 levels and phosphate retention (104, 109). Circulating FGF23 concentrations increase progressively during early and intermediate stages of CKD and can reach levels that are 1,000 times above normal by late stage CKD (110–112). Elevated FGF23 levels in CKD are considered a compensatory mechanism to counteract hyperphosphatemia (113). However, chronically elevated FGF23 levels may become maladaptive to directly stimulate cardiomyocyte growth and induce cardiac hypertrophy in patients with CKD (111).

Soluble klotho levels decline during CKD, which suggests it is a biomarker for CKD diagnosis (114, 115). Studies have shown that the decline in sKl in CKD may be an independent risk factor for CKD-associated cardiac hypertrophy (109). The cardioprotective effects of sKl were investigated using a recognized model of stress-induced cardiac hypertrophy that involves overstimulation by the non-selective β-adrenoreceptor agonist isoproterenol (ISO) (84, 116, 117). Pathological heart growth was induced by ISO in WT mice as reflected by increases in heart size, heart weight indices (heart weight-to-body weight ratio or heart weight-to-tibia length ratio), cardiac fibrosis, and cardiac hypertrophic genes, and these ISO-induced increases were aggravated in *klotho^−/−^* mice (84). Additional studies revealed that klotho deficiency aggravated cardiac hypertrophy in CKD mice, in a manner completely independent of phosphate and/or FGF23 (118). Recombinant klotho ameliorated CKD-associated cardiac hypertrophy without significantly altering serum phosphate and/or FGF23 levels (118). Thus, sKl deficiency is an important risk factor for CKD-associated cardiac hypertrophy independently of the effects of hyperphosphatemia and FGF23.

Injury and stress induce pathological growth and remodeling of the heart. One important regulatory pathway in the development of pathological cardiac hypertrophy involves calcium-mediated activation of the calmodulin-dependent serine-threonine protein phosphatase calcineurin (119). Activated calcineurin dephosphorylates nuclear factor of activated T cells (NFAT) and causes its translocation to the nucleus where it activates cardiac genes involved in hypertrophic growth (119). Calcium influx through multiple TRPC channels is involved in calcineurin signaling and cardiac hypertrophy (120–122). TRPC6 contains NFAT-responsive elements in its promoter, which helps to amplify and sustain cardiac hypertrophic gene expression through a feed-forward circuit (122). Cardiac TRPC channel expression is increased in stress-induced hypertrophic hearts and downregulation of TRPC channels protects against cardiac hypertrophy (123). Thus, TRPC6 is an important mediator of cardiac hypertrophy and may be a therapeutic target. Studies in both ISO-induced cardiac hypertrophy and CKD models supported that α-Klotho protects the heart by downregulating growth factor-driven PI3K-dependent exocytosis of TRPC6 (84, 118).

**Figure 2:**
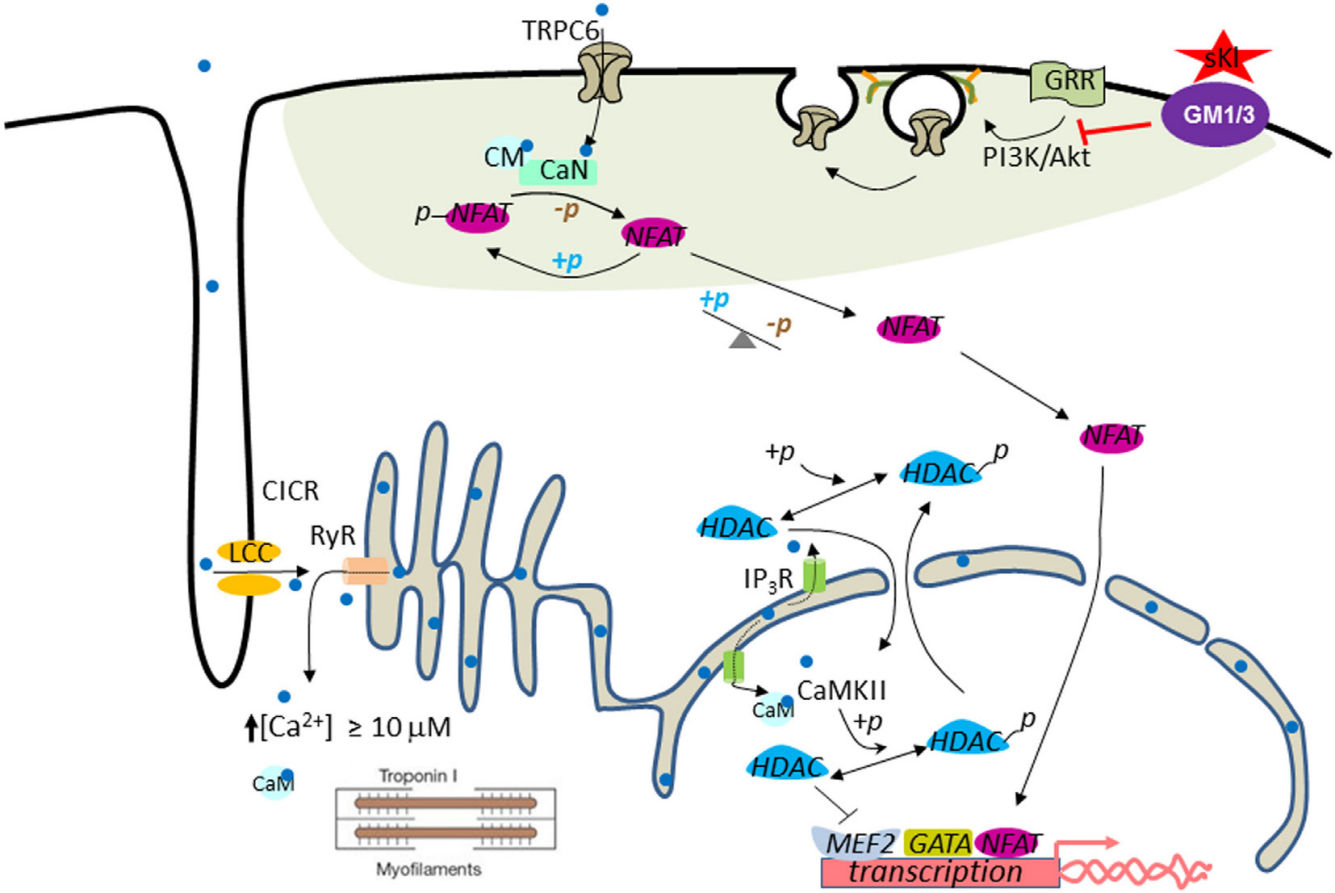
Working model for cardioprotection by soluble klotho (sKl). In the systolic phase, Ca^2+^ (light blue dot) enters through L-type Ca^2+^ channels (LCC) in the T-tube and initiates Ca^2+^-induced Ca^2+^ release (CICR) from ryanodine receptors (RyR). This process results in increased intracellular [Ca^2+^] to ≥10 µM to trigger cardiac contraction (i.e., contractile Ca^2+^). Pathological cardiac remodeling and hypertrophy are triggered by compartmentalized abnormally elevated Ca^2+^ levels, called signaling Ca^2+^. Perinuclear/nuclear Ca^2+^ released from inositol trisphosphate receptor (IP3R) present in the nuclear envelope activates CaMKII–HDAC–MEF2 nuclear signaling cascade. CaMKII, Ca^2+^/calmodulin-dependent protein kinase II; HDAC, histone deacetylase; MEF2, myocyte-enhancer factor-2. Activation of the TRPC6–CaN–NFAT signaling cascade originated from the sarcolemmal TRPC6 channels amplify and sustain cardiac hypertrophic gene expression through a feed-forward circuit [see text for details; calcineurin (CaN)]. Phosphoinositide 3-kinase (PI3K)–Akt signaling is important for exocytotic insertion of TRPC6-containing vesicles. sKl binds to gangliosides GM1 and GM3 (purple ovale) present in the membrane lipid rafts (green–gray shaded area) to inhibit raft-dependent PI3K signaling and TRPC6 channel function. Note that TRPC6 is localized to the lipid raft membrane microdomain.

One important question related to sKl protection of the heart independently of the FGF23–FGF receptor axis is how sKl inhibits PI3K signaling to downregulate TRPC6 in the heart. In other words, what is the receptor that mediates sKl action to inhibit PI3K signaling? Given that FGF23 also activates the PI3K and NFAT signaling cascade and that FGF23 levels are elevated in CKD patients, questions may be raised as to whether cardioprotection observed by administration of sKl is due to binding and neutralization of circulating FGF23. The studies discussed earlier that sKl binds membrane lipid rafts and inhibits raft-dependent PI3K signaling and TRPC6 function provide further support that cardioprotection by sKl is *via* its own membrane receptors and is independent of FGF23 (Figure 2). Finally, other mechanisms for sKl to elicit cellular responses besides *via* binding to gangliosides in lipid rafts, such as direct interaction with IGF-1 receptors and TGF-β receptors are also possible (124, 125).

## Conclusion and Future Perspectives

Identification of membrane lipid rafts and sialogangliosides as receptors have provided new insights into our understanding of how sKl works as a circulating hormone or local autocrine/paracrine factor to exert pleiotropic actions. As in the case of regulation of TRPV5 channels, sKl may target sialic acids to exert its action in different contexts. Other potential mechanisms also exist. Moving forward, it will be important to elucidate the crystal structure of sKl with or without its ligands, which will help with development of smaller active domains of sKl and/or klotho-mimetic for therapeutics. Further understanding of sKl secretion/shedding, regulation, and distribution, as well as handling and pharmacokinetics of endogenous and exogenously administered klotho are also important.

## Author Contributions

GD, JX, S-WA, and C-LH made substantial contributions to the conception and design of the manuscript, were involved in drafting of the work and critical review for important intellectual content, involved in final approval of the version of the manuscript to be published, and agreed to be accountable for all aspects of the work ensuring that all questions related to the accuracy or integrity of any part of the work will be investigated and resolved.

## Conflict of Interest Statement

The authors declare that the research was conducted in the absence of any commercial or financial relationships that could be construed as a potential conflict of interest.
